# Community food program use in Inuvik, Northwest Territories

**DOI:** 10.1186/1471-2458-13-970

**Published:** 2013-10-18

**Authors:** James D Ford, Marie-Pierre Lardeau, Hilary Blackett, Susan Chatwood, Denise Kurszewski

**Affiliations:** 1 Department of Geography, McGill University, Montreal, Canada; 2 Institute for Circumpolar Health Research, Yellowknife, NWT, Canada

**Keywords:** Community food programs, Food security, Arctic Canada, Inuvik, Food banks, Soup kitchen, Traditional foods, Aboriginal, Indigenous

## Abstract

**Background:**

Community food programs (CFPs) provide an important safety-net for highly food insecure community members in the larger settlements of the Canadian Arctic. This study identifies who is using CFPs and why, drawing upon a case study from Inuvik, Northwest Territories. This work is compared with a similar study from Iqaluit, Nunavut, allowing the development of an Arctic-wide understanding of CFP use – a neglected topic in the northern food security literature.

**Methods:**

Photovoice workshops (n=7), a modified USDA food security survey and open ended interviews with CFP users (n=54) in Inuvik.

**Results:**

Users of CFPs in Inuvik are more likely to be housing insecure, female, middle aged (35–64), unemployed, Aboriginal, and lack a high school education. Participants are primarily chronic users, and depend on CFPs for regular food access.

**Conclusions:**

This work indicates the presence of chronically food insecure groups who have not benefited from the economic development and job opportunities offered in larger regional centers of the Canadian Arctic, and for whom traditional kinship-based food sharing networks have been unable to fully meet their dietary needs. While CFPs do not address the underlying causes of food insecurity, they provide an important service for communities undergoing rapid change, and need greater focus in food policy herein.

## Background

Food insecurity has been identified as a major challenge facing Indigenous communities across Arctic Canada, where food systems are comprised of both locally sourced traditional or country foods and externally sourced store foods [1-13]. For some, the difficulty of accessing sufficient, safe, and nutritious food is chronic, particularly for those who do not have an active hunter in the household, have limited access to sharing networks through which traditional foods are distributed, live below the poverty line, and do not have access to permanent shelter [14-16]. Historically, household and community food sharing networks supplied food to the most vulnerable community members. However, such sharing networks are coming under increased stress in the larger settlements; this is a function of demographics, predominance of livelihoods based on the waged economy, acculturation stresses and colonial legacy, in-migration, and transiency in habitation [1,5,17-21]. Moreover, the larger settlements, while increasingly prosperous, have significant pockets of inequality characterized by high and persistent unemployment, poverty, and house overcrowding [22-26]. For these social and economically vulnerable groups, food insecurity is typically chronic and manifests in an inability to access traditional or store foods [19,27].

In response to the growing food security challenge, community food programs (CFPs), including food banks, soup kitchens, and friendship centers, have been developed in some larger Canadian Arctic communities [27]. Such formal food programing is relatively new, and is currently limited to a few regional centers ranging in size from communities like Inuvik and Iqaluit with populations in the range of 3,400-6,700, to Yellowknife with a population >16,000. Little is known about the use of CFPs or the experience and determinants of food insecurity among such users, who are often among the most socially and economically marginalized in communities. In a previous study, and one of the first and only projects focusing on CFPs in an Arctic context, we reported on a case study from Iqaluit, the territorial capital of Nunavut, documenting chronic CFP usage among highly food insecure households who typically do not have access to traditional foods and have limited income for purchasing store foods [27]. The extent to which these findings reflect broader trends in food program usage across Arctic Canada, however, remains unknown, and to our knowledge no other studies have explicitly focused on CFPs in other communities. This is an important research gap, particularly in-light of stressors such as climate change, the rising cost of living, changing sharing networks, population growth, and resource development – all of which have the potential to increase demand for formalized food services, yet have been overlooked in the rapidly expanding Arctic food security scholarship and policy debate [28,29]. This gap in understanding has been identified by policy makers and program coordinators as constraining service planning for this vulnerable segment of the population, and frames the work presented here.

This paper is situated within this deficit in understanding, and uses a mixed methods approach to develop a baseline understanding of CFP usage and associated determining factors, drawing upon a case study from Inuvik, Northwest Territories. In doing so, we build upon and advance our previous work in Iqaluit, Nunavut [27], using a consistent methodology. This allows us to compare similarities and differences between the two communities, a key focus of the discussion, and develop a more in-depth understanding of CFP use in the Canadian Arctic. Given the lack of research on CFPs in northern Canada, comparison is particularly important, and allows us to begin to develop a broad understanding of general trends as well as findings specific to communities / regions.

## Methods

### Inuvik, Northwest Territories

**Figure 1:**
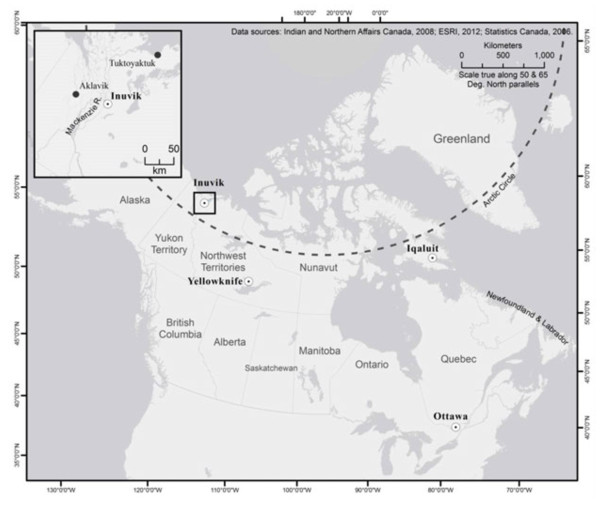
Inuvik, NWT.

Located in Inuvialuit Settlement Region (ISR) of the Northwest Territories (NWT), Inuvik has a population of 3,463, with 63% (n=2,170) identifying as Aboriginal [30], consisting of Inuvialuit, Dene and Metis. The town is located above the Arctic Circle on the Mackenzie River Delta (Figure 1), and was developed during the mid-1950s when the Canadian Government decided there was a need for an administrative centre in the region. The majority of employment opportunities in Inuvik are government-related, or linked to resource development (oil and gas), with some community members still actively engaged in traditional livelihoods (hunting, fishing, trapping). In 2006, the unemployment rate of Inuvik was 11.2%, with average earnings for an Aboriginal person 15 years of age and older being $36,640 [30].

Inuvik’s community food programs (CFPs) include one food bank, one homeless shelter and a *Soup and Bannock* program. The food bank has operated since 2003 and is funded by community events (e.g. bingo, fundraising drives, and donations) (see Table 1 for funding sources for CFPs). It is open for two hours once a week, with users able to pick up non-perishable food purchased by the food bank every other week. Community members also donate foods, including traditional game, which is then distributed to users with the food bags. The homeless shelter opened in 2000 and is currently funded through the Government of the Northwest Territories’ Department of Education, Culture & Employment. The shelter has 21 beds and is open between 18:00 and 10:00, daily. Soup and Bannock lunches are hosted by community organizations during the fall and winter months offering a low cost hot meal for community members.

**Table 1:** Sources of funding for CFPs in Inuvik and Iqaluit

**Community food**	**Funding source**
**program**	
Soup and bannock program (Inuvik)	Unknown
Soup kitchen (Iqaluit)	Government of Canada via City of Iqaluit (~$20,000 annually) donations
Food bank (Inuvik)	Bingo games
	Fundraising
	Donations
Food bank (Iqaluit)	Fundraising
	Donations
	Corporate partnerships
Homeless shelter (Inuvik)	GNWT’s Department of Education, Culture & Employment
	In-kind contributions from the Town of Inuvik (building space)
Tukisigiarvik (Iqaluit)*	Health Canada (~225,000 this year)
	City of Iqaluit (~130,000 this year)
	Qikiqtani Inuit Association (~100,000 this year)
	GN’s Department of Culture and Heritage
	Nunavut Tunngavik Inc.’s Nunavut Harvester Support Program
	In-kind contributions from the GN (building space)

*Note: Tukisigiarvik has over 10 funding sources, so only the main ones are listed here.

The magnitude and extent of food insecurity in Inuvik has not previously been examined. While the town was involved in the Inuit Health Survey (IHS) – a multifaceted health survey conducted in 2007 and 2008 in 33 Inuit communities, with food security a major component – results were not disaggregated at a community-level [31]. While Inuvik is quite different from the five other communities in the ISR, with a larger wage-based economy and population, results from the IHS indicate potential trends, with a prevalence of food insecurity of 43.3% in the ISR as a whole [21]. This is significantly greater than the Canadian average of 9.2%, but less than other Inuit regions, particularly Nunavut (68.8%), with food insecurity correlated with household crowding, income support, public housing, and single adult households [5,21]. Combined with shifting food consumption patterns, and similar to other northern communities, we consider Inuvik to face considerable stress to the food system.

### Data collection

The research involved consultation with territorial level policy makers, local leaders, community members, and northern science bodies to identify research priorities, develop the methodology, conduct the research, and interpret the findings. A mixed methods approach combining qualitative and quantitative methods was developed as a result of this consultation.

#### Photovoice

Photovoice workshops were convened at the beginning of the project to document and examine important factors affecting food security of CFP users, as well as to identify research needs and questions for subsequent interviews. Photovoice involves giving cameras to individuals to photograph their experiences and perceptions of a particular issue, which are then later discussed in an individual or group setting. The method is well-suited to working with Indigenous populations where limits to standard interview-based and survey approaches have been noted, engaging participants as researchers shaping data collection and analysis [19,32-36]. Photovoice participants were recruited from the food bank over a one week period, during which the project was presented to those interested and people were asked to participate in the Photovoice exercise. Seven people agreed to be involved (3 males and 4 females, all unemployed). An introduction to Photovoice was then conducted to explain the research method to participants, discuss ethical concerns surrounding photography, and learn how to take pictures. Participants were asked to answer, through photography, the question: “What aspects of your everyday life affect what you eat and how much you have to eat?” Two workshops to discuss the resulting photographs were then arranged, with participants receiving a $100 gift card for their time. For further details on Photovoice methodology and its use in Canadian Aboriginal contexts, see Lardeau et al. [37], Healey et al. [33], and Castleden et al. [32], and in low income settings, Hofmeijer et al. [34] and Berrang-Ford et al. [38].

#### Food survey

A survey employing fixed-choice and open-ended questions was used to collect standardized data on the experience of food insecurity among CFP users. Each participant was first asked a series of fixed-choice close-ended survey questions covering socio-demographic-livelihood characteristics, food access, and frequency of CFP use. A locally adapted version of the U.S. Department of Agriculture Food Security Module (FFSM), which included 4 questions of the standard 6 item subset of the 12 month FFSM, was then administered. These selected questions are part of the core domains of the food insecurity experience shared across cultures [39], and focus on the experience of not having enough food in the household. The survey is consistent with that used in our previous work in Iqaluit, Nunavut [27], modified for the Inuvik context, therefore permitting comparison with data from this community. Open-ended questions were also asked upon completion of the survey in order to examine and document perceptions of the services offered, the nature of food insecurity experienced, access to country foods, and sharing networks. Surveys were administered in-person by members of the research team with the help of local assistants, and lasted between 15 and 30 minutes. The research team consisted of researchers based at universities in southern Canada (JF, MPL), and based at the Institute for Circumpolar Health Research in Yellowknife (SC, HB) and Inuvik (DK), NWT.

Questions were pre-tested and evaluated by the team to ensure appropriateness and effectiveness. The researchers advertised the study through community flyers posted at the CFPs. Flyers described the research project and stated that participants would be compensated. Consistent with Ford et al. [40], participants selection involved the research team visiting the CFPs during hours of operation, during which users were asked to participate, with sampling continuing through December 2011 until no new users were identified, creating a census of users at this specific period of time. Participants received a $50 CAD gift card for their time, with a response rate > 90%.

#### Other methods

Key informant interviews (n=5) were conducted with personnel at the CFPs to obtain multiple perspectives on utilization of CFPs and challenges faced by users. These interviews were open-ended and conversational, and lasted over an hour in some cases, and were complemented by participant observation during which researchers volunteered at the various CFPs. Finally, to understand how the use of the food bank had fluctuated in the recent years, its usage log was analyzed to assess monthly distribution patterns and to determine if its usage had increased between (usage data was not available for the other programs). It is noteworthy that usage data reflects that people are only allowed to pick up food from the food bank every other week, with usage recorded in bags distributed. A household composed of 1–2 people is allowed 1 bag, 2–3 people 2 bags, 3–4 people 3 bags, and 4 or more people 4 bags.

### Ethics

The research followed ethical norms for working with communities in northern Canada, including obtaining university research ethics board consent from McGill University (REB#: 65–0710), a research license from the Aurora Research Institute (#14804) in the Northwest Territories, eliciting written informed consent, and ensuring confidentiality of participants.

### Analysis

The Photovoice pictures were analyzed during workshops held in Inuvik. Each picture was projected onto a screen and the group was asked to discuss the meaning of the photographs and how they interpreted each. Participants told stories about their personal experiences accessing food, which resulted in the generation of messages that were attached to each photo. Participants identified which photos seemed to fall under similar themes, and were then asked to organize the pictures according to these themes. The group collectively decided on the final themes and messages around the selected photographs: these themes, photos, and quotes in turn, helped structure the presentation of results below. A note taker recorded all comments made about each photograph. Each photographer had the choice to remain anonymous.

Data from the surveys were analyzed in SPSS 15.0. Basic descriptive statistics were used to describe the sample population, responses to each question, and to ascertain the distribution of responses by age, sex, occupation, and hunting behavior. Chi-squared (χ^2^) analysis and Fisher exact tests were performed to test for significant differences between participant characteristics and responses to questions, as well as test for significant differences between sample characteristics and the general population of Inuvik (derived from the 2006 census). Open-ended questions from the survey were used to examine participants’ experiences and perceptions of CFPs in Inuvik, while key informant interviews were used to document the perspectives of those running the respective programs. Coding was used to sort qualitative answers in content-related categories by using non-automated frequency counts and conducting latent content analysis. Average weekly bag counts were used to assess seasonal patterns in food bank usage. A simple linear regression using an Analysis of Variance (ANOVA) was used to test for a trend in food bank usage between February 2009 and July 2012, the period over which data are available. All statistical results were considered significant at the 95% confidence level. All results were discussed with CFP users and key informants.

## Results

In this section we profile socio-demographic characteristics of CFP clientele in Inuvik and determinants of use, and finish by comparing the Inuvik data with Iqaluit results from our previous work [27]. The results documented here draw upon key themes identified in the Photovoice workshops and emerging from the analysis of surveys, were discussed with CFP users and personnel on completion of the work, and are accompanied with photographs and quotes selected by participants to illustrate the experience and determining factors of CFP use.

### Food program users are more likely to be housing insecure, female, middle aged (35–64), unemployed, Aboriginal, and lack a high school education

Fifty-four community members participated in the survey, with food program users more likely to report Aboriginal identity (p<0.01, Fisher’s exact test) compared to the general Inuvik population (98% v 63%). Food program users were more likely to be female (p=0.04, χ^2^), with 65% of participants female compared to 51% in the general Inuvik population. The majority of participants were born in Inuvik (74%). Of those not from Inuvik, most were from another community in the Northwest Territories (90%) and had lived in Inuvik from more than 5 years (91%). Food program users were less likely to be employed (p<0.01, χ^2^) compared to the general Inuvik population, and the majority were homeless or lived at the shelter at the time of the survey (35%), 31% lived with extended family of friends, 12% in a two-parent household, and 12% were single parents. Food program users were significantly less likely to report having a high school diploma or higher (p<0.01, χ^2^). For example, while 69% of the general Inuvik population have a high school certificate or higher, only 26% of food program users have this level of education. Finally, food program users reflect a middle-age cross-section of the Inuvik population, and were less likely to be among young (≤35yrs) and older (≥65yrs) age cohorts (p=0.01, Fisher’s exact test). Full response characteristics are given in Table 2.

**Table 2:** Socio-economic demographic data for study participants from Inuvik and Iqaluit

**Indicators**	**Study participants,**	**Inuvik, NWT**^**1**^
	**n (%)**	**N (%)**
**Population**	54	3463
Reporting Aboriginal or Inuit identity^2^	53 (98)	2170 (63)
Male	19 (35)	1700 (49)
Female	35 (65)	1765 (51)
**Age group**		
18-24	4 (8)	365 (11)
25-34	6 (11)	610 (18)
35-44	17 (32)	460 (13)
45-54	16 (30)	525 (15)
55-64	9 (17)	340 (10)
65-74	1 (2)	145 (4)
**Born in Inuvik**	39 (74)	*Data not available*
**Employment rate (3)**	29 %	71%
**Source of income (all family types)**		
Employment income	15 (31)	*Data not available*
Government transfer payments	24 (49)	*Data not available*
Other income sources	10 (20)	*Data not available*
**Housing (2)**		
Husband-Wife or Common Law	6 (12)	645
Single parent household	6 (12)	240
Living with extended family or friends	16 (31)	*Data not available*
Living alone	3 (6)	*Data not available*
Homeless or shelter	18 (35)	*Data not available*
Other living arrangements	3 (6)	*Data not available*
**Education (4)**		
Proportion (%) with no certificate, diploma or degree	74	*Data not available*
Proportion (%) with high school diploma or more	26	69

^1^Based on 2001 census http://www12.statcan.ca/census-recensement/2011/dp-pd/prof/details/page.cfm?Lang=E&Geo1=CSD&Code1=6101017&Geo2=CD&Code2=6101&Data=Count&SearchText=inuvik&SearchType=Begins&SearchPR=61&B1=All&Custom=&TABID=1 Accessed October 15^th^ 2012.

2 2006 Stats can census.

3 http://www.statsnwt.ca/community-data/Pamphlets/Inuvik.pdf.

4 2009 data http://www.statsnwt.ca/community-data/Profile%20PDF/Inuvik.pdf.

### The food bank is widely used

**Figure 2:**
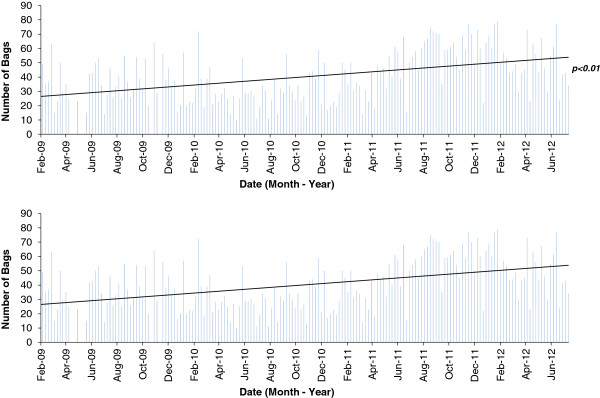
Number of bags distributed by the food bank on a weekly basis in Inuvik, February 4th 2009 – July 12th 2012.

Between February 4, 2009 and July 11, 2012, 7,133 food bags were distributed, with an average of 41 bags distributed per week (Figure 2). March has the lowest average number of bags distributed per week (33 bags), while November had the highest average number of bags distributed per week (53 bags). The maximum and minimum number of bags distributed in a week was 79 and 10, respectively. A significant increase (p<0.01) in the number of bags distributed was observed from February 2009 to July 2012. We do not have data on Inuvik population for this time period, although between 2006 and 2011, population declined by 2.2%.

### Food program users are chronically food insecure

“Do we really have choices? We can’t get [traditional] foods at the store, so we have to get the meat from the south. The [traditional] food at the community freezer is too expensive, so we can’t access it. The food from the food bank is useful, but it’s always the same thing. Sometimes we get to choose between canned vegetables and canned beans…”

**Figure 3:**
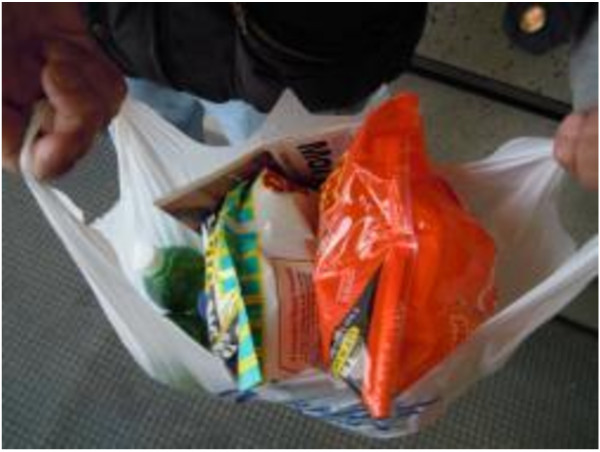
Food choices.

**Figure 4:**
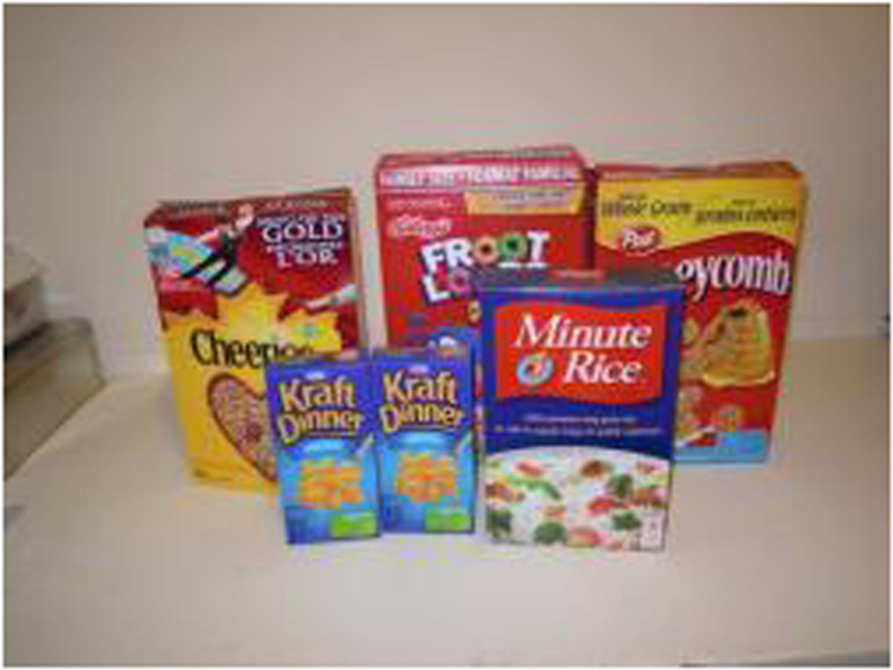
Food preferences.

**Figure 5:**
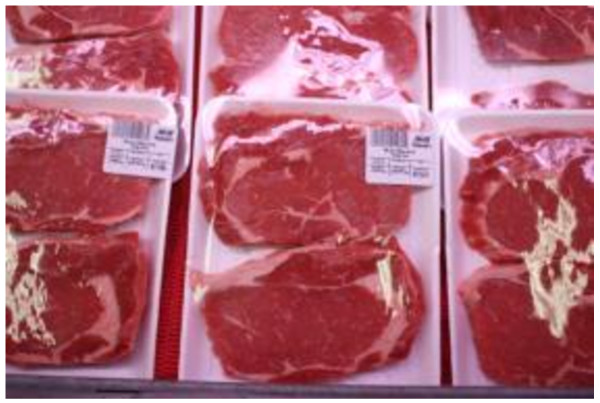
High cost of living and food affordability.

More than two thirds of the participants (70%) had experienced times in the last year when there was not enough food at home and it was not possible to access more. Coping strategies at these times included: switching to less preferred and lower quality foods (70%), reducing portions for oneself (64%), reducing portions for others in the household (45%), selling things to access money for food (42%) and sending household members to eat elsewhere (28%). These findings are consistent with the Photovoice workshops, where participants widely reported the limited availability of traditional foods and high cost of nutritious foods leading participants to eat less healthy foods (Figures 3, 4, 5).

Participants discussed the difficulties obtaining nutritious foods and also noted that many craved high fat, greasy foods. When these items could be afforded, they were generally purchased before nutritious foods (Figure 3). Participants discussed the difficulties of having children who only crave junk food, and how this was often the cheapest option to feed them (Figure 4). As one participant noted when describing a photograph of a bottle of pop: “*There is no nutritional value in this, but it tastes good. It’s not good for you. It is banned in the school but for some kids, this is their breakfast with a bag of chips, that’s all they eat…”.*

There was also discussion in the Photovoice workshops around the Canada Food Guide, which was not followed by participants, mostly because the cost associated with the purchase of fruits, vegetables and dairy product made them inaccessible. Finally, participants in the workshop described the fact that because they could not afford traditional foods at the store, they would sometimes purchase imported southern meats, which were not what they really wanted, and found them to be prohibitively expensive (Figure 5).

### Traditional foods are not consistently available to food program users

The surveys documented that around half of the participants had a hunter in their household, and the majority of participants (87%) had a hunter in their extended family. Traditional foods (TFs) were available on a daily basis for 18% of participants, every week for 20%, every month for 26% and occasionally for 36%. TFs were mostly obtained through family and friends (70%) and during community feasts (11%). Very few participants obtained TF through their own means (6%).

**Figure 6:**
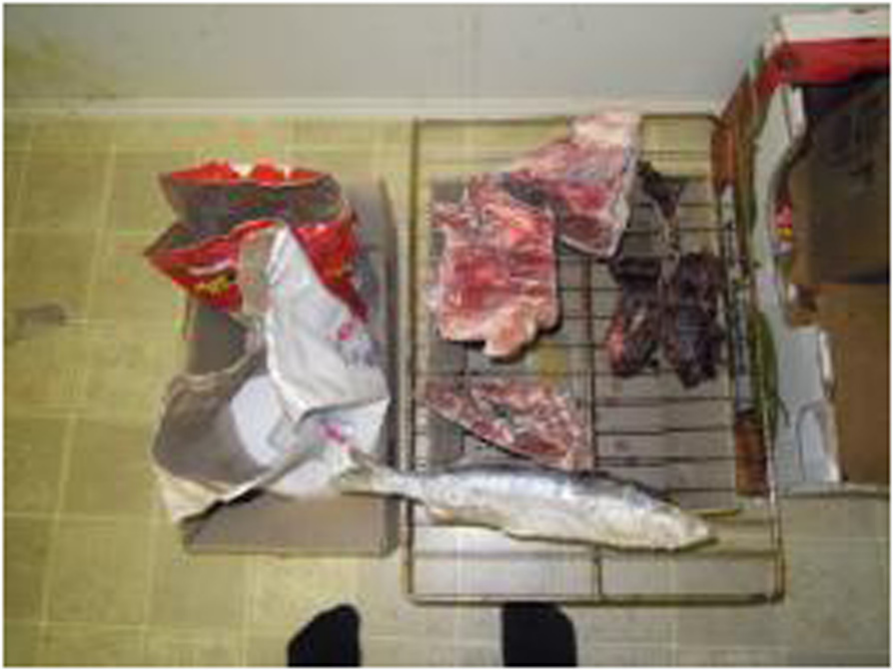
Traditional food availability.

**Figure 7:**
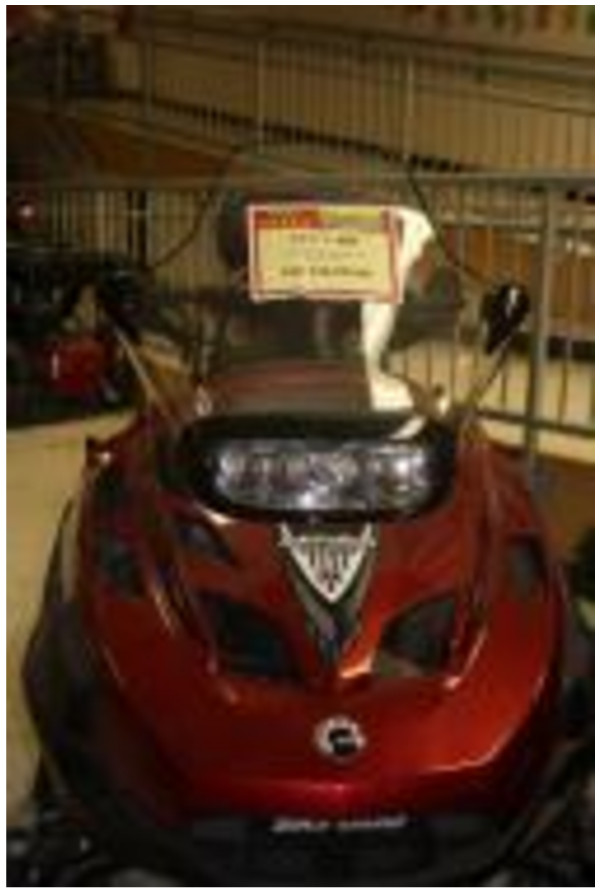
High cost of hunting and fishing.

**Figure 8:**
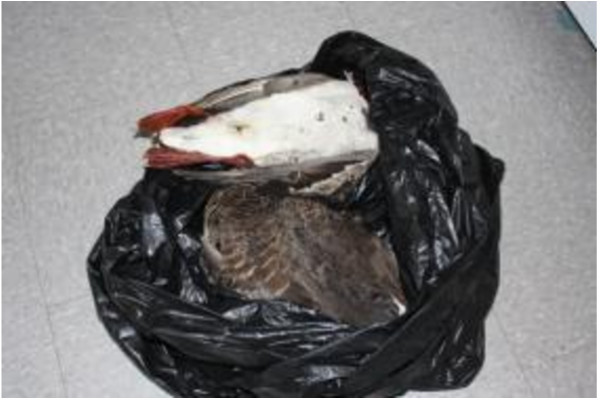
Knowledge on traditional food preparation.

**Figure 9:**
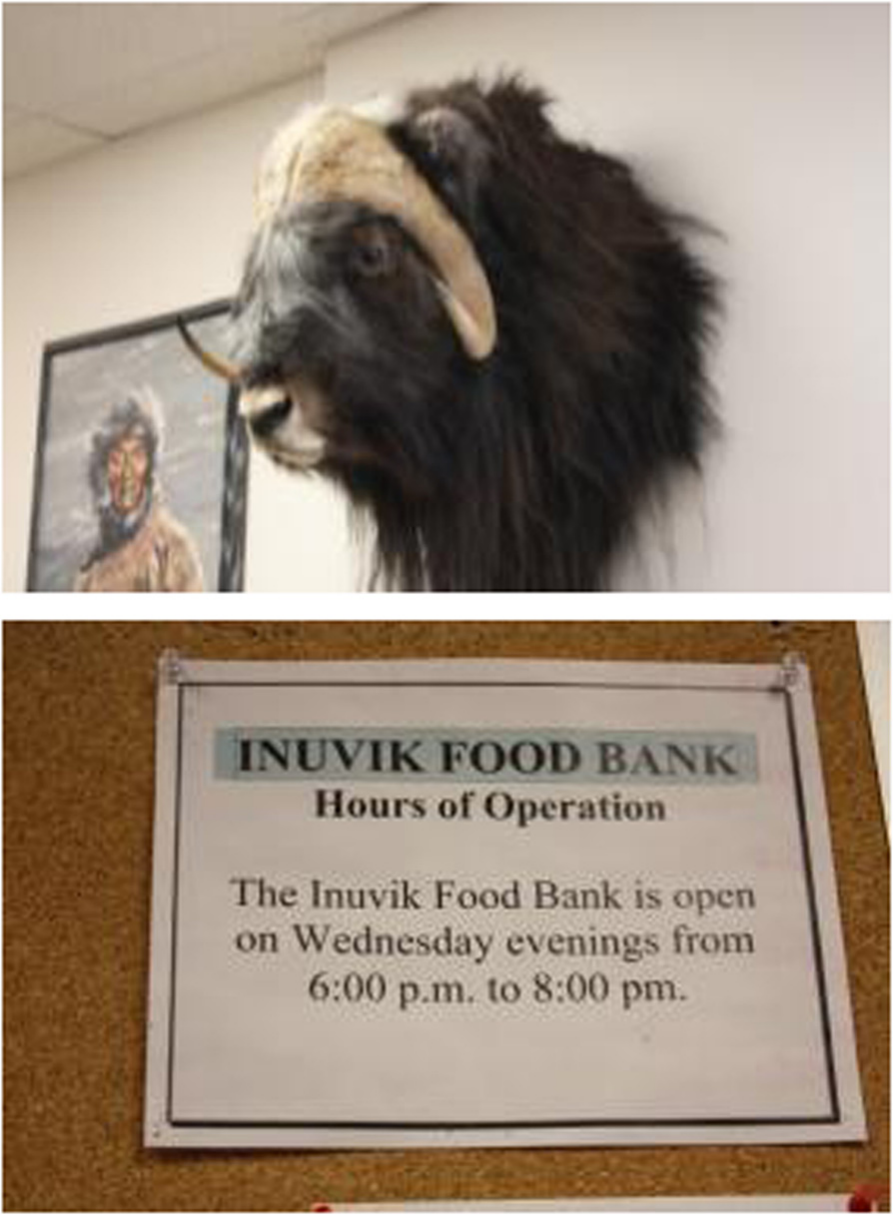
Changing availability of traditional foods.

During the Photovoice workshops and for the open ended survey questions, participants discussed the importance of TF and how the high cost to obtain them made access difficult (Figure 6). Participants expressed the importance of such foods, noting preference for caribou which was described as a staple for most of the users’ lives. The lack of availability of TF, the lack of hunting equipment, the high price of gas in Inuvik combined with the fact that most participants did not own a vehicle or snowmobile, reduced access to TF. This is a problem chronically faced by CFP users (Figure 7). Participants reported having family members or friends who could supply them with TF occasionally (80%), which was highly valued and appreciated. However, the loss of knowledge on how to prepare TF was identified as a challenge for some; one Photovoice participant explained not knowing how to prepare geese which had been given to her by family members (Figure 6 and 8). Some resorted to purchasing TF when available, such as musk-ox or reindeer, although the cost was very high for these items (Figure 9).

### Community food programs are an important source of food for those in-need

**Figure 10:**
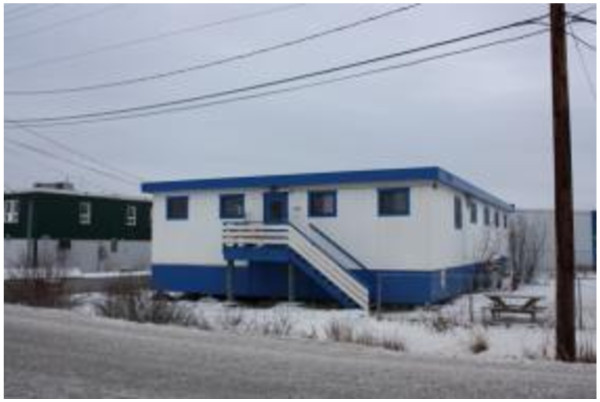
Importance of the homeless shelter.

The majority of participants reported using the food bank (93%), less than half the soup and bannock program (48%) and 39% the homeless shelter (Figure 9 and 10). The food bank was used at least once a month by 62% of participants. Most participants used the soup and bannock program occasionally throughout the year, with 67% reporting using it two times a month.

The Photovoice workshops highlighted the importance of community food programs in Inuvik for food access, particularly for homeless participants. Discussions described the importance of the food bank for both the homeless who lived in make-shift shelters on the outskirts of town and for those who were ‘couch surfing’ or moving from house-to-house in Inuvik. Participants were grateful for the services offered but also expressed their wish for more variety and a freezer where TF could be stored (Figure 5). The winter months were described as being more difficult for many in terms of accessing TF, with community feasts (celebrations or funerals) described as the only source of access to TF for some at this time. During the workshops, participants described how using such services could help them save a bit of money on their food budget, which was considered important for them as well as for other community members.

### Many factors influence a sense of vulnerability

The survey and interview questions aimed to explore some of the factors that led to a sense of vulnerability to food insecurity. Whereas 39% of participants felt that there was no specific time during the year that was more difficult to have enough food, 18% considered late fall (Nov-Dec) to be the most difficult time, followed by winter (14%), early fall or spring (9%) and during the summer (7%). Around one third (31%) of participants thought that their situation was equally difficult throughout the month to access enough food, whereas 38% stated that the most difficult time was when services providing food were closed or when they were between social security payments.

When asked if the number of people living in their house fluctuated during the year, 58% of participants said yes, Of these participants, 70% stated that winter was the time of year when numbers fluctuated the most and when the greatest amount of people were living with them. Also, 15% who had fluctuating numbers of people in their houses said that the time they had the most people was when friends and family had nowhere to go.

**Figure 11:**
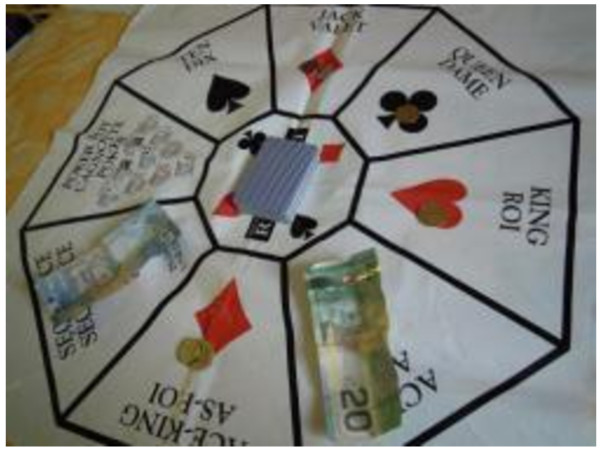
Addiction and food insecurity.

**Figure 12:**
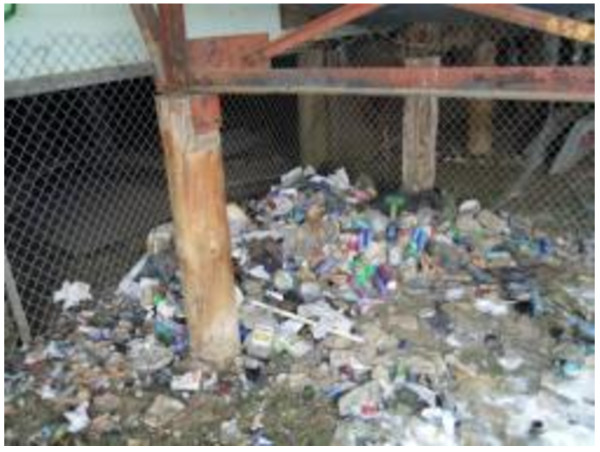
Addictive behavior needs to be understood in the context of acculturative stresses affecting northern populations.

**Figure 13:**
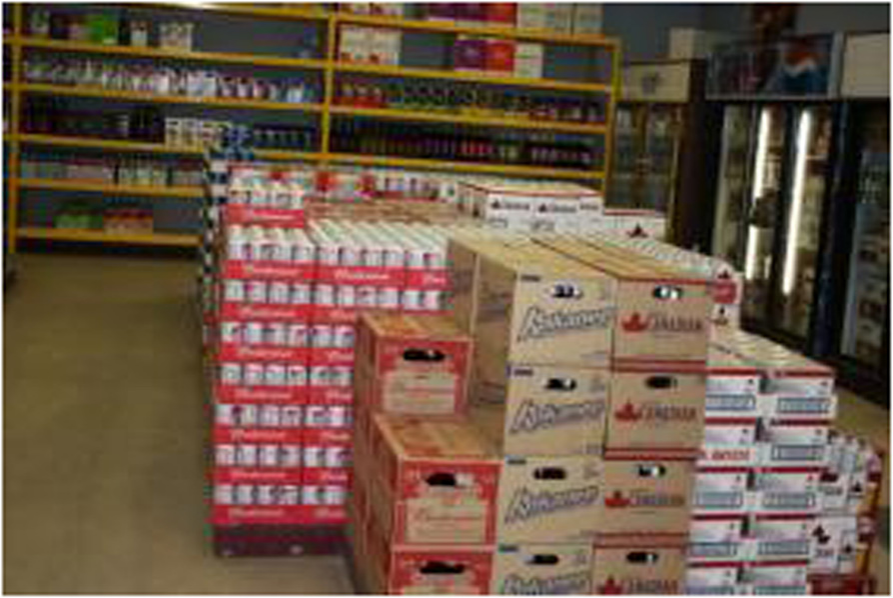
Addiction can take away money from food purchasing.

Finally, the Photovoice workshops also highlighted addictions and ability to hold down a job as important challenges for many. Some survey participants discussed the wide-spread issue of addictions in and around Inuvik and how available funds were often spent on alcohol or gambling (Figures 11, 12, 13). Gambling was also described as being a widespread problem in the community, with which some having dealt with for years.

### There are similarities and differences between Inuvik and Iqaluit

The clientele of Inuvik CFPs less frequently noted experiencing conditions of food insecurity, with 70% reporting experiencing times in the last year when it was not possible to obtain enough food in the home compared to 89% in Iqaluit. They were also less likely to report a lack of employment than in Iqaluit (p<0.01, χ^2^), with fewer also noting that their income was rarely or never enough for their needs (46% vs 57%). CFP users in Inuvik were twice as likely to report being homeless, while in Iqaluit users were twice as likely to live in two-parent households. There were no significant differences in age, gender, or education of users in the two communities. Comparison of rates of food bank usage was not possible due to differences in data collection (Inuvik recording bags handed out and Iqaluit households supported), and different operating times (Inuvik individuals permitted only every other week, Iqaluit every week).

## Discussion

This paper develops preliminary insights on the use of community food programs (CFPs) in Inuvik, Northwest Territories. To our knowledge this is the first study to focus on CFPs in the NWT, and only the second in the Canadian Arctic, developing important baseline information on those community members highly vulnerable to food insecurity. As such, this work helps to fill in gaps in the scholarship which has typically focused on smaller more traditional communities, and avoided examining food security for high risk groups.

Users of CFPs in Inuvik are typically housing insecure, are not employed, lack a high school education or higher, are female, and report Aboriginal identity. These findings are not surprising, capturing those who have been identified as being at highest risk of food insecurity in the Canadian north [5,11,21,41-44]. The results are more interesting however, when compared with our previous work with food program users in Iqaluit [27] – part of a larger project supported through ArcticNet seeking to understand and characterize the food insecurity of high risk population in regional centres in the Canadian North – and can help us begin to sketch out a broad profile of who is using CFPs, how often, why, and how this use is changing over time, along with identifying community specific characteristics. Both Iqaluit and Inuvik are gateway communities to the regions in which they are located (Figure 1), provide important administrative functions, have large Aboriginal populations (~60% in both locations), combine a waged economy with elements of traditional livelihoods, and are undergoing rapid changes in livelihoods and economy linked to resource development and integration into national/global economies. They also differ in terms of population size, Aboriginal groups represented, and characteristics of the local environment. Comparing the results from both communities, six findings are of particular interest.

Firstly, chronic use of CFPs was widely reported by participants in both Iqaluit and Inuvik, the majority of who report using at least one of the food programs once a month or more. For many, CFPs are the only reliable source of food, with users housing insecure and lacking regular employment. These characteristics are broadly consistent with CFP users in southern Canada, although in Inuvik and Iqaluit the clientele tends to be middle aged, have less education, and are primarily Aboriginal [45]. It is noteworthy that in both communities very few people ≥65 years of age were observed using the CFPs. This is consistent with qualitative research on Arctic food systems, where supplying elders with food, particularly TF, is highly valued [18,46,47]. The predominance of middle-aged users, particularly those in their 50s, could also be linked to the past experience of residential schools and associated trauma in this age group [48], while food programs targeting the general population typically focuses on children (e.g. breakfast programs) and pregnant women (e.g. Canada Prenatal Nutrition Program), and often overlook those who are middle aged.

Secondly, users frequently linked challenges they faced in obtaining sufficient food to addictive behavior and an inability to hold down a job – an observation made in other food security studies in northern communities [19,49,50]. In Inuvik, like Iqaluit, the primary Aboriginal clientele of CFPs have witnessed significant acculturative stresses over the past half century associated with relocation from smaller settlements and semi-permanent hunting camps beginning in the mid-1950s, collapse of livelihoods dependent on the fur trade, environmental dispossession, and associated and often forced cultural assimilation (e.g. through residential schools). Such context frames many challenges facing Canada’s northern Aboriginal populations and are described in detail elsewhere [48,51-56]. While we didn’t investigate issues of abuse, criminal history, addiction and their socio-cultural context in-depth due to the sensitive nature of these questions and preliminary nature of the research, our observations and key informant interviews indicate these challenges to widely experienced among CFP users, and emphasizes the need for food policy to be more broadly integrated into wellness initiatives.

Thirdly, while the role of traditional foods differentiates the experience of northern CFP users from those in southern Canada, for many participants in Inuvik and Iqaluit such foods were inconsistently and unpredictably available, and not typically procured by users themselves but sourced from sharing networks. Approximately two thirds of CFP users in both communities reported having access to sharing networks for traditional foods (TFs), yet it was frequently noted that TF access was irregular, infrequent, and in many cases becoming more challenging as the number of full-time hunters decreases, costs of hunting increase, and with disruption caused by climate change. Even participants with a hunter in the household were not more likely to report having greater access to TF, likely a function of cost constraints which make hunting difficult for those without access to financial resources. As such, the experience of CFP users differs from that of northern populations in general, where Huet et al. [5], reporting on results from the Inuit Health Survey, find that the presence of a hunter in the household acts as a significant buffer to food insecurity.

Fourthly, participants in both communities reported utilizing a similar bundle of coping mechanisms to deal with the challenge of obtaining sufficient food. Over 40% of participants in both communities reported selling household items to access food, and over half of respondents in Iqaluit reported sending household members to eat elsewhere when food was hard to obtain (compared to 28% in Inuvik).

Fifthly, in both communities, CFPs are widely depended upon by users and act as essential services, particularly in-light of the rapid transitions in food systems being experienced in the larger Canadian Arctic communities which are stressing the traditional and store food components of the food system [46,47,57-59]. CFPs have emerged over the last decade in response to these challenges, and are a recent development in the North, where historically those in-need were supported through household and family kinship networks in smaller more homogeneous communities than today. Users are therefore highly vulnerable to disruptions to CFP operations. For instance, the Inuvik food bank was vandalized in early September 2012, only re-opening in October, and significantly limited options for those without regular food access.

Finally, the characteristics of CFP clientele also differ in key respects. Users in Iqaluit reported more frequent and severe experiences of food insecurity. This likely reflects the higher degree of unemployment among Iqaluit participants and higher costs of living in the community, with other studies reporting greater prevalence of food insecurity and other indicators of socio-economic hardship in Nunavut compared to other northern regions [21,23,41,60]. It is noteworthy however, that homelessness was more frequently noted among Inuvik participants.

These findings have particular relevance in-light of increased attention to food policy in the Arctic, and especially given the recent report of the United Nations Special Rapporteur on the Right to Food who noted the need for Canada to develop a national Right to Food strategy after his visit to the North in May 2012 [12]. A number of initiatives seek to address food insecurity including the recently revamped food mail program (Nutrition North), which provides a retail subsidy that has received much attention since implementation in the last year and required adjustments in the early implementation. Adjustments in recent months demonstrate the complexities of implementing policy to support food security in remote northern communities, and need for ongoing and proactive policy research in this area.

Other policy areas of interest are those not specifically food related, but have direct impacts on food availability, specifically policy related to wildlife management, such as bans and restrictions to caribou hunting. Wildlife management and policy in the Northwest Territories is developed under co-management boards, a proactive model that recognizes the jurisdiction of land claim and self-government agreements. CFPs have been largely invisible in policy debate on food security across the North, yet their chronic use suggests that they are more than just a stop-gap measure, providing an essential community service for vulnerable populations. While the rapid economic development being experienced in northern Canada associated with resource development may help alleviate some of the negative socio-economic conditions underlying an inability to access food, the food security challenge goes beyond financial constraints, linked to acculturative stress, historical trauma, and changing socio-cultural systems. CFP users are among the most affected by these broader stresses, and least positioned to benefit from new economic openings, with a need for broad-level policies focusing on overall-health and well-being, and targeting the root causes of vulnerability. CFPs don’t address these root causes, although they do provide social networks and sense of well-being for users, and can form part of broader programing.

The importance of TFs has been widely promoted in northern food policy, offering a nutritionally and culturally important source of food. Research, for instance, indicates better overall health and lower food insecurity among those with high TF consumption [5,21,61]. Yet while CFP users value TFs and recognize their importance, access is limited. As respondents noted in the study here and Iqaluit, health messages focusing on TF consumption are of little value to those who do not have access to these food and are at times a source of frustration. Therefore, efforts to provide TFs through community food programs were frequently noted by participants to be important. More broadly, research and policy debate across the north has focused on ways to assist those procuring traditional foods, who face a number of barriers related to cost and training. Recommendations have included enhancing harvester support to enable harvesters to afford equipment and gas costs, training for younger generations on how to procure and work with traditional foods safely, including the documentation and promotion of traditional land skills, reduced freight rates for shipping traditional foods between communities, and investment in community freezers [15,47,50,62-67]. Despite this, an evidence base on successful interventions for policies strengthening the traditional susbsitence sector and links to food security is lacking.

As was learned during this study, CFPs that do not receive government funding must look to other sources to support their services. The food bank is almost entirely funded through bingo games in the community (Table 1), which were identified during the Photovoice discussions as being a means through which community members spend their money and become vulnerable to food insecurity. The cycle of poverty continues for these community members and questions the use of such initiatives as a means of supporting CFPs. Alternatively, in Iqaluit, the food bank and soup kitchen rely on donations, while the Tukisigiarvik friendship centre receives funds from multiple sources, including the federal government. While historically, household sharing networks would ensure distribution of traditional foods to vulnerable community members – and still do today to an extent, as documented in this study – they have come under increasing strain with population growth, economic development, and the growth of waged employment [15,17,40,68-70]. Many participants described food sharing as unpredictable, frequently leaving them in challenging situations when food was not available. Social welfare provides important access to financial resources for individuals, including income assistance and housing support (Table 3), while poverty alleviation programs have a strong emphasis on enhancing food security (Table 4). Notwithstanding, welfare was described by many as insufficient, particularly for those with large families and/or single parent households. For the homeless users of CFPs however, and similar to Iqaluit, access to welfare is limited, and is compounded by the lack of housing security and access to sharing networks. These are typically chronic users of CFPs, for whom there are few alternative sources of food, and further underscores the importance of food programs.

**Table 3:** Social welfare program in Inuvik and Iqaluit

**Social safety net programs**	**Details**
GNWT income support (Inuvik)	$39.6 million*
GN income assistance (Iqaluit)	$40.0 million**
GNWT public housing (Inuvik)	$15.8 million*
GN public housing (Iqaluit)	$118.0 million**

*GNWT’s 2012/13 budget (GNWT, 2013).

**GN’s 2012/13 budget (GN, 2013).

GNWT (2013). *Building on the Strengths of Northerners: A Strategic Framework toward the Elimination of Poverty in the NWT*. Accessed 16 June 2013 from http://www.assembly.gov.nt.ca/_live/documents/content/13-06-06TD%2097-17(4).pdf.

GN (2013). *Main Estimates 2013–2014*. Prepared by the Department of Finance. ISBN # 978-1-55325-230-6. Accessed 16 June 2013 from http://www.finance.gov.nu.ca/apps/authoring/dspPage.aspx?page=index-budgets.

GN (2011). *The Makimaniq Plan: A Shared Approach to Poverty Reduction*. Accessed 16 June 2013 from http://www.makiliqta.ca/sites/default/files/the_makimaniq_plan_final_eng_20.12.11.pdf.

**Table 4:** Povert reduction plans in Inuvik and Iqaluit

**Poverty reduction plan**	**Content**
*Building on the Strengths of Northerners: A Strategic Framework toward the Elimination of Poverty in the NWT* (Inuvik)	Released in 2013
	Five priorities:
	• Children and Family Support (Breakfasts for Learning Program and the Canada Prenatal Nutrition Program as examples of current initiatives promoting healthy children and families)
	• Healthy Living and Reaching our Potential (healthy eating initiatives by Health and Social Services as a health promotion example of current initiatives promoting healthy living and reaching our potential)
	• Safe and Affordable Housing (soup kitchens and food programs as supporting efforts to reduce homelessness)
	• Sustainable Communities (Community Harvesters
	• Assistance Program, Inuvialuit Hunters Assistance Program, NWT Growing Forward Program, and Small Scale Foods Program examples of harvesting initiatives promoting sustainable communities)
	• Integrated Continuum of Services
	Food security appears as a component of some of the five priorities. Two objectives related to food security initially appeared in the former poverty reduction document entitled *Poverty Free NWT: An Anti-Poverty Strategy for the Northwest Territories*. These objectives fell under the Healthy Children and Families theme, and included “all young children have access to the food they need for healthy growth and development” and “all youth have the food they need to be healthy and reach their physical and cognitive potential.” However, these food security-specific objectives have been removed in the final poverty reduction plan.
*The Makimaniq Plan: A Shared Approach to Poverty Reduction* (Iqaluit)	Released in 2011
	Six themes:
	• Collaboration and Community Participation
	• Healing and Wellbeing
	• Education and Skills Development
	• Food Security
	• Housing and Income Support
	• Community and Economic Development
	Food security is specifically one of the six themes.
	The objectives for the Food Security theme include:
	1) The establishment of a “Nunavut Food Security Coalition”
	2) Enhancement of healthy breakfast and lunch programs in schools
	3) Increased support for community-driven food security initiatives
	The Nunavut Food Security Coalition has since been established, and is creating the Nunavut Food Security Strategy. The Strategy will be complete in June 2013.

Limitations of the study include the need to caution that work represents user trends and determining factors documented during December Inuvik during 2011 (i.e. sampling bias), with users potentially varying by year and month, and in comparison with the Iqaluit fieldwork conducted in 2010. Temporal variations in food security could be pronounced given the importance of environmental conditions in affecting the availability of traditional foods, and also reflecting seasonal employment, yet have not been examined in previous food security studies using food surveys. This requires further investigation both in relation to CFP usage and food security more broadly in northern Canada. The use of questions derived from the USDA food security module in our survey also bring limitations, in terms of the question items used which may lack construct validity in northern Aboriginal settings [5,61,71]. Their use here reflects the need to compare with other studies conducted in northern Canada – for instance through the Inuit Health Survey [5,8,9,21,60,72] – and generally in Canada, all of which are based on the USDA module. Like others, however, we advocate for the development of an Aboriginal specific food security survey. Other limitations include the fact that we do not breakdown the analysis by cultural group of the Aboriginal population in Inuvik (First Nations, Inuit, and Metis), consistent with our study in Iqaluit.

## Conclusion

This paper reports on a preliminary investigation of who is using community food programs (CFP) in Inuvik NWT, and determining factors. Comparing the findings with a comparable study in Iqaluit, the results contribute towards a nascent understanding of food insecurity and CFP use among some of the most vulnerable community members in northern Canada, who have been overlooked in the burgeoning Arctic food security scholarship. We caution that the results provide only a snapshot of use at a particular point in time and represent the first investigation of CFPs in the community. The exploratory work identifies a number of areas for future research, including: examining usage trends at different times of the year to further investigate seasonal influences; investigating in greater depth differences in use, experiences, and determining factors among user characteristics, specifically by different Aboriginal groups; further exploring the role of sharing, kinship and social relations in affecting CFP usage; and examining policy development to enhance the food systems of CFP users.

## Competing interests

The authors declare no competing interests.

## Authors' contributions

JF and MPL conceptualized and wrote the paper. DK, HB, and MPL conducted fieldwork. SC assisted with project conceptualization, fieldwork and writing. All authors read and approved the final manuscript.

## Pre-publication history

The pre-publication history for this paper can be accessed here:

http://www.biomedcentral.com/1471-2458/13/970/prepub
